# An Effortful Approach to Social Affiliation in Schizophrenia: Preliminary Evidence of Increased Theta and Alpha Connectivity during a Live Social Interaction

**DOI:** 10.3390/brainsci11101346

**Published:** 2021-10-13

**Authors:** Lilian Y. Li, Jason Schiffman, Derek K. Hu, Beth A. Lopour, Elizabeth A. Martin

**Affiliations:** 1 Department of Psychological Science, University of California, Irvine, CA 92697, USA; lilian.y.li@northwestern.edu (L.Y.L.); jeschiff@uci.edu (J.S.); 2 Department of Psychiatry and Behavioral Sciences, Northwestern University, Chicago, IL 60611, USA; 3 Department of Biomedical Engineering, University of California, Irvine, CA 92697, USA; hudk@uci.edu (D.K.H.); blopour@uci.edu (B.A.L.)

**Keywords:** schizophrenia, negative symptoms, social affiliation, functional connectivity, theta oscillations, alpha oscillations

## Abstract

People with schizophrenia often experience a profound lack of motivation for social affiliation—a facet of negative symptoms that detrimentally impairs functioning. However, the mechanisms underlying social affiliative deficits remain poorly understood, particularly under realistic social contexts. Here, we investigated subjective reports and electroencephalography (EEG) functional connectivity in schizophrenia during a live social interaction. Individuals with schizophrenia (*n* = 16) and healthy controls (*n* = 29) completed a face-to-face interaction with a confederate while having EEG recorded. Participants were randomly assigned to either a Closeness condition designed to elicit feelings of closeness through self-disclosure or a Small-Talk condition with minimal disclosure. Compared to controls, patients reported lower positive emotional experiences and feelings of closeness across conditions, but they showed comparably greater subjective affiliative responses for the Closeness (vs. Small-Talk) condition. Additionally, patients in the Closeness (vs. Small-Talk) condition displayed a global increase in connectivity in theta and alpha frequency bands that was not observed for controls. Importantly, greater theta and alpha connectivity was associated with greater subjective affiliative responding, greater negative symptoms, and lower disorganized symptoms in patients. Collectively, findings indicate that patients, because of pronounced negative symptoms, utilized a less efficient, top-down mediated strategy to process social affiliation.

## 1. Introduction

Human beings are fundamentally motivated for social affiliation—the desire to engage in and maintain close relationships with other people [1]—yet it is woefully lacking in some people with schizophrenia. Diminished motivation for social affiliation is one of the most common negative symptoms of schizophrenia [2]; it remains relatively stable across the course of illness and has deleterious impacts on functional outcomes across domains [3,4]. Despite the clear clinical importance of social affiliative deficits, our understanding of the processes that contribute to this impairment is limited, as in part reflected by the insufficiency of current treatments in improving affiliative motivation in schizophrenia [3].

In the service of developing targeted treatments and improving outcomes, considerable efforts have been made to explicate the neural mechanisms underlying deficient social affiliation in schizophrenia. Specifically, past research has focused on parsing the component parts of this complex construct through the use of simplified laboratory tasks. This work has revealed a number of components altered in schizophrenia, including face perception, salience attribution, emotion regulation, and mentalizing [5,6,7,8]. In particular, the large body of literature on facial affect processing has consistently shown hypoactivity in regions and processes related to early visual processing (e.g., fusiform gyrus and N170 for structural encoding of facial features) and emotion processing (e.g., amygdala and N250 for evaluating facial emotions) [9,10,11]. Possibly to compensate for these impairments, patients correspondingly display hyperactivity in regions related to higher-order visual processing (e.g., cuneus, parietal lobule, and superior temporal gyrus) [10,11]. While informative, it still remains unclear how these regional abnormalities interact and collectively contribute to social affiliative deficits. As social affiliation involves the concerted coordination of multiple bottom-up (e.g., face perception) and top-down (e.g., mentalizing) processes, a better understanding of the functional interactions between brain regions is needed.

Electroencephalography (EEG) functional connectivity may be a particularly suitable tool for delineating such neural dynamics. Specifically, it can capture the synchronous neuronal oscillations in different frequency bands that subserve brain communications both locally (i.e., high frequencies such as beta and gamma) and between larger scale networks (i.e., low frequencies such as delta, theta, and alpha) [12,13,14,15]. To date, only a handful of studies have employed EEG functional connectivity to examine face perception in schizophrenia (see [16] for a review). Findings indicate an overwhelming reduction in connectivity across all frequencies except for the delta band [17,18,19,20,21]. Perhaps more informative for understanding social affiliative deficits, Popov and colleagues [17] examined alpha connectivity as facial expressions of happiness and fear dynamically unfolded over time. Compared to controls, patients showed reduced alpha connectivity in the central region, including the somatosensory cortex that is responsible for embodied simulation (i.e., making inferences through the production of the corresponding affective state) [22]. At the same time, patients (vs. controls) showed increased alpha connectivity in the frontal and occipital regions that are indicative of an exaggerated top-down control [23]. Hence, there is preliminary evidence to suggest that schizophrenia is associated with altered functional connectivity in a number of frequency bands with nuanced topographical characteristics.

Although the aforementioned studies provided valuable insights on the abnormal connectivity networks associated with social affiliative deficits in schizophrenia, they are limited by their use of overly simplified face stimuli. Critically, capturing the dynamic influences on social affiliation requires ecologically valid assessments of actual interactions, which has been an increasing focus in recent years [5]. In the laboratory setting, studies have typically employed imagined interactions (e.g., playing games with computerized partners) and role plays (e.g., acting out fake scenarios with the experimenter). These interaction-based studies predominately focused on subjective reports, none of which examined functional connectivity, and have yielded mixed results. While some studies observed comparable affiliative responding between patients and controls, including emotional experience, affiliative reaction to the interaction partner (e.g., partner liking and closeness), and social motivation [24,25], others found experience and motivation deficits in patients [26,27,28]. However, within the schizophrenia group, there is converging evidence that deficits are more pronounced with increasing negative symptom severity [24,25,26,28]. Nevertheless, the employed interaction paradigms often lack real-life qualities (e.g., turn-taking and responding to ongoing reactions from the partner), and some even engendered negative reactions that may hinder affiliative bonding (e.g., [29]). Hence, the extent to which existing interaction-based findings can speak to affiliative functioning in schizophrenia patients is quite limited, and we do not yet know the neural underpinnings of social interactions.

To test the feasibility of quantifying EEG-based functional connectivity in a realistic social context, this pilot study examined connectivity across five frequency bands (i.e., delta, theta, alpha, beta, and gamma) in response to a live, face-to-face interaction task among patients with schizophrenia and healthy controls. Participants were randomly assigned to either an Interpersonal Closeness Generation condition or a Small-Talk condition, both involving asking and answering a pre-set list of questions between interaction partners. The Closeness condition was designed to elicit affiliative bonding, whereas the Small-Talk condition lacked this feature [30]. Therefore, comparing the two conditions crucially allowed us to disentangle the subjective and neural processes most relevant for social affiliation.

## 2. Materials and Methods

### 2.1. Participants

Participants consisted of 16 clinically stable outpatients with schizophrenia and 30 healthy controls. Patients with schizophrenia were primarily recruited from a Veterans Affairs Hospital (*n* = 12). Some patients (*n* = 4) and all controls were recruited via advertisements posted in the community and on Craigslist. Schizophrenia patients met the diagnostic criteria for schizophrenia (*n* = 8) or schizoaffective disorder (*n* = 8), confirmed by the Structured Clinical Interview for DSM-5 (SCID-5) [31]. Controls were free of any psychiatric disorders or first-degree relatives with a psychotic disorder. Additional selection criteria for all participants included (a) an absence of substance abuse disorder within the past 6 months, (b) an IQ > 70 based on the National Adult Reading Test (NART) [32], (c) a sufficient fluency in English, and (d) an absence of any neurologic event or disease (e.g., loss of consciousness for more than 10 min or stroke). One control participant was excluded from analysis due to insufficient EEG data. Demographic and clinical information of the included participants is shown in Table 1.

### 2.2. Procedure and Materials

After providing informed consent, participants were assessed with the SCID-5. Participants then completed a social interaction task while having EEG recorded (see Section 2.3 for details on EEG acquisition). Immediately following the task, participants completed several measures in the order listed below; these measures assessed the interaction, affiliative reactions towards the partner (i.e., liking and closeness), and future motivation to engage in a similar task. Upon completion, participants received monetary compensation. This study was approved by the relevant Institutional Review Boards.

#### 2.2.1. Psychosis Symptoms

Psychosis symptoms were assessed using the Scale for the Assessment of Negative Symptoms (SANS) [33] and the Scale for the Assessment of Positive Symptoms (SAPS) [34]. Symptom ratings were made by an experienced clinical psychologist (EAM). Prior studies suggest that SANS and SAPS are best represented by a four-factor structure consisting of two positive symptom dimensions (i.e., reality distortion and disorganization) and two negative symptom dimensions (i.e., inexpressivity and apathy/asociality) [4]. The four subscale scores were thus calculated by averaging the high-loading items outlined in Kotov et al. [4].

#### 2.2.2. Social Interaction Task

Participants were randomly assigned to either an Interpersonal Closeness Generation condition or a Small-Talk condition [30]. For both conditions, participants were told that they were going to participate in an “enjoyable sharing game” with another study participant, who in fact was a confederate. There were two participating confederates, both were female in their early 30 s with light skin, dark eyes, dark hair, and similar build. Then, the confederate was brought into the room and seated directly across from the participant, both configured with the EEG equipment. During the task, the participant and the confederate asked and answered 30 pre-set questions divided into three sessions, with each session lasting for approximately 12 min. The Closeness condition (8 patients and 14 controls) involved questions designed to elicit feelings of closeness through personal disclosure (e.g., “What is one of your most treasured memories?”; “Tell your partner what you like about him/her. Be very honest this time saying things that you might not say to someone you’ve just met.”). As participants progressed through the task, the questions became increasingly more intimate and personal, both within each session and over the three sessions. In contrast, the Small-Talk condition (8 patients and 15 controls) involved questions designed to gain superficial knowledge of each other with minimal disclosure (e.g., “If you could invent a new flavor of ice cream, what would it be?”; “How often do you get your hair cut?”). To create a similar social environment across participants, the confederate was blind to group status and gave the same answer to each question for all participants. After completing the social interaction task, the confederate was escorted from the room.

#### 2.2.3. Post-Interaction Ratings

The interaction evaluation measure [35] is comprised of 12 items from four subscales: emotional experience (e.g., “How much did you enjoy the sharing game?”; α = 0.63), engagement (e.g., “How much did you feel you influenced the interaction?”; α = 0.71), quality (e.g., “How much did you consider the interaction to be smooth and natural?”; α = 0.66), and disclosure (e.g., “How much did you feel you disclosed to your game partner?”; α = 0.82). Participants rated the extent to which they agreed with each item on an 8-point scale (1 = not at all; 8 = very much).

Partner liking was assessed using a version of the Interpersonal Judgement Scale (IJS) [36]. The IJS is comprised of two items (“How much do you like your game partner?” and “How much would you like to participate in a future sharing game with your partner?”) rated on a 7-point scale (1 = dislike very much; 7 = like very much; α = 0.90).

Feelings of closeness were assessed using the Inclusion of Other in the Self (IOS) scale [37]. The IOS scale is comprised of seven pairs of circles representing Self and Other that overlap in increasing degrees (1 = nonoverlapping; 7 = maximal overlap). Participants selected the pair of circles that best described their relationship with their interaction partner.

Future motivation was assessed with one item (“How likely would you be to participate in a similar sharing game in another study?”) rated on a 7-point scale (1 = extremely unlikely; 7 = extremely likely).

### 2.3. EEG Recording and Preprocessing

For participants only, continuous EEG activity was recorded across the three sessions using the ANT-Neuro system (ANT-Neuro, Hengelo, The Netherlands). Recordings were from 31 Ag/AgCl channels (FPz, FP1, FP2, Fz, F3, F4, F7, F8, FC1, FC2, FC5, FC6, Cz, C3, C4, T7, T8, CPz, CP1, CP2, CP5, CP6, Pz, P3, P4, P7, P8, POz, Oz, O1, O2) embedded in a cap and placed according to an expanded 10/20 system [38]. Channels were referenced online to CPz and data were sampled at 1000 Hz.

All signal processing was conducted offline in MATLAB using customized scripts and the EEGLAB toolbox [39]. Signal processing was carried out following established pipelines [40,41] and broadly included filtering, removing and correcting artifacts, and retaining brain-related activities. Specifically, EEG data were first downsampled to 500 Hz and the DC offset was removed from each channel. A high-pass, 2nd-order Butterworth filter was then applied at 0.1 Hz. The 60 Hz line noise was removed using the cleanLineNoise function, which used a sliding window to adaptively estimate and subtract the line noise component [40]. Next, artifactual channels were removed, identified as those (a) containing significant periods of flat signal, (b) containing extreme amplitudes with a robust z-score greater than 5, (c) having maximum correlation with all other channels less than 0.4 (computed in 1 s non-overlapping windows) for more than 1% of the time windows, (d) correlating less than 0.75 with their predicted activity estimated from other channels (computed in 5 s non-overlapping windows) for more than 4% of the time windows, or (e) containing high frequency noise to signal ratio with robust z-score greater than 5 [40]. All artifactual channels were replaced by whole head spline interpolation, and data were re-referenced to the common average.

Next, Artifact Subspace Reconstruction (ASR) [42] was applied to correct for significant noise bursts. ASR is a principal-component-analysis-based (PCA-based) technique in which data within a 500 ms sliding window (window step = 250 ms) were PCA-decomposed. Noisy PCs, defined as those with variance greater than 20 *SD* above that of the clean portions of the data, were removed and the data were then reconstructed from the remaining PCs. Further, time windows were removed if more than 25% of the channels contained high-power artifacts, defined as greater than 7 *SD* above the clean power estimates in the channel. The data were again re-referenced to the common average so that the sum across all channels was zero. Sections of data with amplitudes greater than 500 µV in a sliding window of 500 ms (window step = 250 ms) were then removed.

Lastly, independent component analysis (ICA) was implemented to retain brain-related components only. These components were defined as (a) having greater probability to be brain than artifacts according to an automatic IC classifier (ICLabel) [43], (b) having residual variance (i.e., the difference between IC′s scalp projection and the projection of fitted equivalent current dipole) less than 15%, and (c) having fitted dipole location within the brain.

### 2.4. Functional Connectivity

After preprocessing, continuous 8 s epochs of clean EEG data were extracted from each participant. One control participant was removed due to insufficient data (i.e., fewer than 20 epochs). On average, participants contributed 134.49 epochs (*SD* = 53.38). The number of epochs was not significantly related to connectivity strength for any frequency bands (all *p*s > 0.38). Epochs were first bandpass filtered separately at 1.5–4 Hz (delta), 4–8 Hz (theta), 8–13 Hz (alpha), 13–30 Hz (beta), and 30–50 Hz (gamma) using an FIR filter. The filter order was five times the lower passband frequency, and the transition zone was 25% of the lower passband frequency. The analytic signal was obtained by taking the Hilbert transform of the filtered EEG signal for each epoch and channel.

For each epoch, the functional connectivity between each channel pair was estimated using the weighted phase lag index (wPLI) in the following way [44,45]:

(1) $${\mathrm{wPLI}}_{xy}=\frac{\left|\frac{1}{n}\sum_{t=1}^{n}\left|imag\left(S_{xyt}\right)\right|sign\left(imag\left(S_{xyt}\right)\right)\right|}{\frac{1}{n}\sum_{t=1}^{n}\left|imag\left(S_{xyt}\right)\right|}$$

where $S_{xyt}$ is the cross-spectrum between two channels *x* and *y* at time *t*. The wPLI calculates the mean signum of the imaginary component of the cross-spectrum, weighted by the magnitude of the imaginary component. Thus, cross-spectrum elements that are further away from the real axis have a larger influence on the estimate of connectivity, thereby limiting the influence of small noise perturbations changing the “true” sign of the imaginary component [45]. The wPLI ranges from 0 to 1. A wPLI of 0 reflects no phase coupling or coupling at 0 mod π, which may be indicative of volume conduction. On the other hand, a wPLI of 1 reflects consistent phase coupling at a value different from 0 mod π. The functional connectivity of each participant was calculated by averaging the wPLI across epochs, yielding one 31-by-31 adjacency matrix (i.e., 31-by-31 channel pairs). The connectivity strength of a channel was subsequently calculated as the sum of all wPLI values connected to that channel.

### 2.5. Statistical Analyses

All analyses were performed using R [46]. First, to examine differences in post-interaction ratings, an ANOVA of Group (Control vs. Patient) X Condition (Small-Talk vs. Closeness) was conducted for each measure. Next, to examine differences in functional connectivity, a multilevel model (MLM) of Group (Control vs. Patient) X Condition (Small-Talk vs. Closeness) X Region (Frontal vs. Central vs. Occipital) was conducted for each frequency band, with random intercepts of participants and channels. The frontal region consisted of FP1, FPz, FP2, Fz, F3, F4, F7, and F8; the central region consisted of FC5, FC1, FC2, FC6, Cz, C3, C4, T7, T8, CP1, CPz, CP2, CP5, and CP6, and the occipital region consisted of Pz, P3, P4, P7, P8, POz, Oz, O1, and O2. All significant interaction effects were followed up with post hoc contrast analyses.

For the patient group only, the association of functional connectivity with symptom and post-interaction ratings was examined in an MLM of Rating X Region (Frontal vs. Central vs. Occipital) for each frequency band, with random intercepts of participants and channels. Simple slopes displaying the association for each region were subsequently calculated.

## 3. Results

### 3.1. Post-Interaction Ratings

Ratings for interaction evaluation, partner liking, closeness, and future motivation are shown in Table 2. For all ratings, the interaction of group and condition was nonsignificant (all *p*s > 0.14). Thus, analyses were rerun without the interaction. There was a significant main effect of condition for ratings of engagement, *F*(1,42) = 5.35, *p* = 0.026, and partner liking, *F*(1,42) = 5.33, *p* = 0.026; participants in the Closeness condition reported greater ratings compared to those in the Small-Talk condition (engagement: *d* = 0.71; partner liking: *d* = 0.70). There was a significant main effect of group for ratings of emotional experience, *F*(1,42) = 5.71, *p* = 0.021, and closeness, *F*(1,42) = 5.21, *p* = 0.028; patients reported lower ratings than controls (emotional experience: *d* = 0.77; closeness: *d* = 0.72). Neither conditions nor groups differed significantly in ratings of quality, disclosure, and future motivation (all *p*s > 0.48, *d*s < 0.23). Thus, across patients and controls, the Closeness (vs. Small-Talk) condition elicited greater levels of engagement in participants and greater levels of liking towards the partner. Patients (vs. controls) experienced lower levels of positive emotional experiences and feelings of closeness towards the partner across conditions.

### 3.2. Functional Connectivity

**Figure 1:**
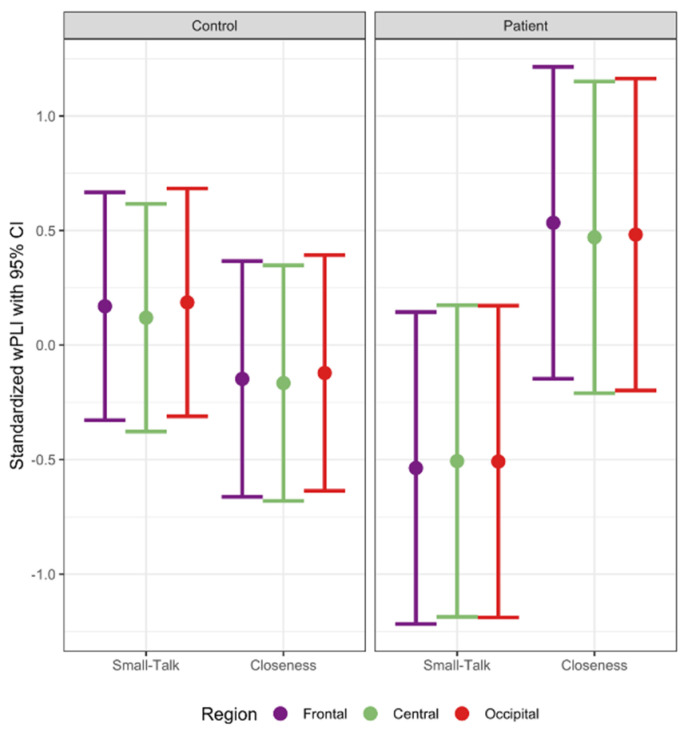
Theta connectivity strength.

**Figure 2:**
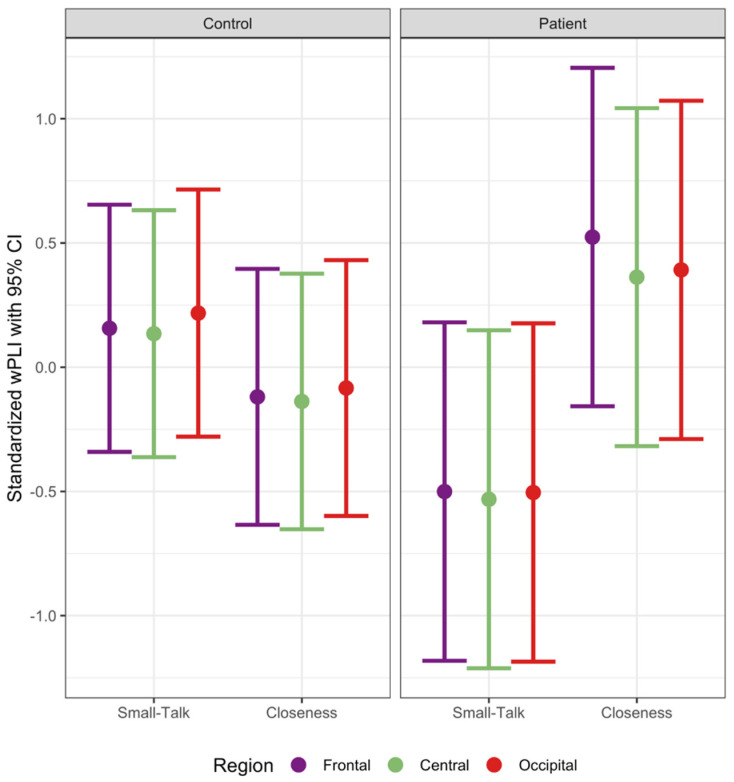
Alpha connectivity strength.

Average adjacency matrices are shown in the Supplementary Materials. For the theta band, there was a significant three-way interaction of group, condition, and region, *F*(2, 1314.00) = 5.66, *p* = 0.0036 (see Figure 1). For controls, Closeness and Small-Talk conditions did not significantly differ in theta connectivity for all regions (all *p*s > 0.39). However, patients in the Closeness condition showed greater theta connectivity in the frontal region compared to patients in the Small-Talk condition, β (*SE*) = 1.07 (0.49), *t*(41.15) = 2.18, *p* = 0.035. In addition, patients in the Closeness (vs. Small-Talk) condition showed marginally greater theta connectivity in central and occipital regions (central: β (*SE*) = 0.98 (0.49), *t*(41.06) = 1.99, *p* = 0.053; occipital: β (*SE*) = 0.99 (0.49), *t*(41.12) = 2.02, *p* = 0.050). A significant three-way interaction was also observed for the alpha band, *F*(2, 1314.00) = 3.67, *p* = 0.026 (see Figure 2). Similarly, whereas controls did not significantly differ between conditions in all regions (*p*s > 0.41), patients in the Closeness (vs. Small-Talk) condition showed greater alpha connectivity in the frontal region, β (*SE*) = 1.02 (0.49), *t*(41.26) = 2.09, *p* = 0.043, and marginally greater alpha connectivity in central and occipital regions (central: β (*SE*) = 0.89 (0.49), *t*(41.11) = 1.82, *p* = 0.076; occipital: β (*SE*) = 0.90 (0.49), *t*(41.22) = 1.83, *p* = 0.075). For other frequency bands (i.e., delta, beta, and gamma), neither conditions nor groups differed significantly in any of the regions (all *p*s > 0.10). Thus, patients displayed greater theta and alpha connectivity in response to the Closeness (vs. Small-Talk) condition, particularly in the frontal region.

### 3.3. Association of Functional Connectivity with Symptom and Post-Interaction Ratings in Patients

Analyses were restricted to theta and alpha bands that showed an effect of interest, and the standardized simple slope estimates are shown in Table 3. With respect to psychosis symptoms, greater alpha connectivity across regions was marginally associated with lower disorganization. Additionally, greater frontal alpha connectivity was marginally associated with greater inexpressivity. While nonsignificant, the association between alpha connectivity and apathy/asociality was in the medium-effect range. Thus, patients with greater negative symptoms and lower disorganized symptoms displayed a trend for greater alpha connectivity, particularly in the frontal region.

With respect to post-interaction measures, greater theta and alpha connectivity across regions was associated with greater partner liking. Greater theta connectivity in the frontal and occipital regions was also marginally associated with greater feelings of closeness. A positive association of theta and alpha connectivity with interaction evaluation and future motivation was also observed, although failed to reach statistical significance. Specifically, an effect of at least medium magnitude was observed for theta connectivity with emotional experience, engagement, quality, and future motivation as well as for alpha connectivity with engagement, quality, disclosure, and future motivation. The effects were generally strongest in the frontal region. Thus, greater theta and alpha connectivity, particularly in the frontal region, was generally associated with better experience of, and motivation for, the interaction.

## 4. Discussion

The present study sought to elucidate the nature of social affiliative deficits in schizophrenia by examining subjective reports and EEG functional connectivity in response to a live social interaction. To precisely isolate subjective and neural correlates of social affiliation, patients with schizophrenia and healthy controls completed one of the two interaction conditions that varied in the extent of affiliative bonding (i.e., Closeness vs. Small-Talk). Results demonstrated that the Closeness condition was indeed more conducive for the formation of a social bond than the Small-Talk condition, showing greater levels of engagement and partner liking across patients and controls. While the Closeness (vs. Small-Talk) condition elicited greater subjective affiliative responding in patients commensurate with that of controls, as supported by a lack of significant interaction effect between group and condition, patients showed a general reduction in positive emotional experiences and closeness across conditions. This finding corresponds well with prior studies showing that patients, although on average report lower positive affect and higher negative affect in their day-to-day lives [47], do reap emotional benefits from daily social interactions that typically involve close others (e.g., family members) [5]. Therefore, subjective affiliative deficits might be consequent to a broad affective abnormality, rather than specifically linked to the generation of close relationships.

Nevertheless, the functional connectivity results reveal that patients’ normative increase in subjective affiliative responses to the Closeness (vs. Small-Talk) condition comes at a cost. Specifically, whereas connectivity strength did not differ between conditions for controls, patients in the Closeness condition showed a global increase in theta and alpha connectivity compared to patients in the Small-Talk condition. Critically, in patients, greater connectivity in both theta and alpha bands was associated with greater affiliative experience and motivation, and greater alpha connectivity was associated with greater negative symptoms and lower disorganized symptoms. As such, the current connectivity findings could be interpreted as patients exerting more effort in generating affiliative responses to circumvent obstacles posed by negative symptoms. This finding is in line with prior studies on facial affect processing observing top-down mediated compensatory responses in patients, such as elevated frontal and occipital alpha connectivity [17], and hyperactivity in higher-order visual processing regions [10,11]. Perhaps not surprisingly, this effortful neuronal synchronization was impaired by symptoms of disorganization, which, at its core, reflects a fragmentation of perceptual and cognitive coordination [48,49].

What might be the functional meaning of increased theta and alpha connectivity? Both frequency bands have been implicated in top-down control and coordination of interregional communications, but they carry out these functions in distinct ways [15,23,50]. In particular, theta activity plays a role in directing attention to information of motivational significance, especially towards object features (e.g., crinkled eyes in a smile), as well as integrating bottom-up sensory information with top-down memory representations [50,51,52,53]. On the other hand, alpha activity is involved in information gating by inhibition, including selective attention and memory retrieval [23,54,55,56]. Considering the functions of theta and alpha bands together, the current findings seem to imply that when processing affiliative social interaction, patients with schizophrenia undertook a more cognitive, feature-based strategy, whereas controls relied more on an intuitive, configural-based strategy. Specifically, because of the pronounced negative symptoms (e.g., social inattentiveness), patients engaged in more top-down modulation of attention to salient features, which were then matched with relevant memory templates to comprehend the affiliative context. Controls, however, engaged in more configural processing of social information (e.g., perceiving facial emotions as a whole rather than individual features) that allowed for an efficient inference of affiliation. Although speculative, our interpretation aligns with the extensively documented configural face processing impairment in schizophrenia [57,58]. Nevertheless, replication studies are needed before firm conclusions can be drawn.

Despite offering intriguing insights on the altered neural dynamics associated with deficient social affiliation in schizophrenia, this pilot study has several limitations. First, because of the small sample size, we only had sufficient power to detect large effects. Consequently, the null findings have to be interpreted with caution. For example, patients (vs. controls) showed numerically greater theta and alpha connectivity for the Closeness condition and numerically lower theta and alpha connectivity for the Small-Talk condition, both of large magnitude (βs around 0.5 to 0.6). It is therefore possible that under mundane social contexts, patients failed to generate adequate neuronal synchrony to build social relationships while expanding exaggerated neural effort when the affiliative demand was high. It will be important for studies with larger sample sizes to replicate the current findings and to further probe the influence of important covariates such as gender, age, race, and medication. Second, similar to previous interaction-based studies [24,25], we only included female interaction partners because both men and women generally react more favorably to women [59]. It remains unclear how gender and other demographic characteristics (e.g., age and race) of the interaction partner may influence participants’ responses. Lastly, while significant efforts were made to ensure a realistic social interaction, we lacked measures of functional outcomes and thus cannot conclusively determine whether the observed findings relate to functioning in the community. In addition, aside from abnormalities that transpire during the course of an interaction, other barriers may exist that limit the initiation of social engagement (e.g., few social opportunities and fear of rejection) [60,61]. Future studies may wish to comprehensively investigate how multiple sources of social barriers interact to precipitate social affiliative deficits in schizophrenia.

## 5. Conclusions

In conclusion, this is the first study to investigate subjective reports and EEG functional connectivity in schizophrenia during a live social interaction. Findings indicate that patients with schizophrenia relied on a more effortful, top-down mediated approach to process social interaction so as to generate subjective affiliative responses. This increased neural effort was associated with greater negative symptom severity, thereby indicating a mechanistic pathway from negative symptoms to social affiliative deficits. Further, the present study provided initial support for the feasibility of studying connectivity among patients under a realistic social context. Further real-world applications (e.g., in patients’ natural environments) would be a fruitful avenue for understanding and improving diminished social affiliation in schizophrenia.

## Figures and Tables

**Table 1:** Demographic and clinical characteristics of participants.

Characteristics	Patient *n* = 16	Control *n* = 29	Test Statistics
Female *n* (%)	7 (43.75)	18 (62.07)	χ^2^(1, *N* = 45) = 0.76, *p* = 0.38, *V* = 0.13
Age *M* (*SD*)	49.31 (13.33)	42.17 (13.16)	*t*(43) = 1.73, *p* = 0.090, *d* = 0.55
Race *n* (%)			*p* = 0.20 (two-tailed Fisher′s exact test), *V* = 0.34
Black	5 (31.25)	2 (6.90)	
White	7 (43.75)	14 (48.28)	
Latinx	2 (12.50)	5 (17.24)	
Other	2 (12.50)	8 (27.59)	
Education *M* (*SD*)	13.62 (2.12)	15.48 (1.99)	*t*(43) = −2.92, *p* = 0.0055, *d* = 0.93
Psychosis symptoms *M* (*SD*)			
Reality distortion	0.96 (0.85)	-	
Disorganization	0.16 (0.32)	-	
Inexpressivity	0.34 (0.61)	-	
Apathy/Asociality ^a^	1.26 (1.39)	-	

^a^ The item on sexual interest and activity was not assessed.

**Table 2:** Mean (standard deviation) of post-interaction ratings.

Measures	Patient		Control	
	Closeness *n* = 8	Small-Talk *n* = 8	Closeness *n* = 14	Small-Talk *n* = 15
Interaction evaluation				
Emotional experience	6.41 (1.01)	6.44 (1.66)	7.14 (0.61)	7.08 (0.60)
Engagement	6.71 (1.20)	5.79 (2.09)	6.67 (1.18)	5.78 (0.95)
Quality	7.08 (0.92)	6.79 (1.72)	6.76 (1.12)	7.16 (0.74)
Disclosure	6.81 (1.16)	7.06 (1.15)	6.86 (0.93)	7.03 (0.83)
Partner liking	6.75 (0.71)	6.25 (1.16)	6.93 (0.18)	6.43 (0.78)
Closeness	4.12 (1.55)	3.75 (1.91)	5.21 (0.97)	4.60 (1.24)
Future motivation	6.75 (0.46)	6.12 (1.12)	6.36 (1.34)	6.73 (0.59)

**Table 3:** Standardized simple slope (standard error) estimates for the association of functional connectivity with symptom and post-interaction ratings in patients.

Measures	Theta			Alpha		
	Frontal	Central	Occipital	Frontal	Central	Occipital
Psychosis symptoms						
Reality distortion	0.15 (0.27)	0.16 (0.27)	0.16 (0.27)	−0.11 (0.27)	−0.10 (0.27)	−0.13 (0.27)
Disorganization	−0.27 (0.26)	−0.26 (0.26)	−0.27 (0.26)	−0.47 (0.24) †	−0.44 (0.24) †	−0.46 (0.24) †
Inexpressivity	0.20 (0.27)	0.17 (0.27)	0.17 (0.27)	0.48 (0.25) †	0.38 (0.25)	0.39 (0.25)
Apathy/Asociality	0.22 (0.27)	0.21 (0.27)	0.21 (0.27)	0.37 (0.26)	0.29 (0.26)	0.29 (0.26)
Interaction evaluation						
Emotional experience	0.31 (0.26)	0.30 (0.26)	0.31 (0.26)	0.18 (0.27)	0.17 (0.27)	0.19 (0.27)
Engagement	0.33 (0.26)	0.30 (0.26)	0.30 (0.26)	0.31 (0.26)	0.27 (0.26)	0.29 (0.26)
Quality	0.34 (0.26)	0.31 (0.26)	0.33 (0.26)	0.44 (0.25)	0.39 (0.25)	0.42 (0.25)
Disclosure	0.22 (0.27)	0.20 (0.27)	0.21 (0.27)	0.35 (0.26)	0.29 (0.26)	0.33 (0.26)
Partner liking	0.54 (0.24) *	0.50 (0.24) †	0.53 (0.24) *	0.47 (0.24) †	0.43 (0.24) †	0.45 (0.24)
Closeness	0.44 (0.25) †	0.42 (0.25)	0.44 (0.25) †	0.28 (0.26)	0.30 (0.26)	0.31 (0.26)
Future motivation	0.43 (0.25)	0.39 (0.25)	0.42 (0.25)	0.39 (0.25)	0.35 (0.25)	0.36 (0.25)

Note: df = 14; † *p* < 0.10; * *p* < 0.05.

## Data Availability

Dataset and reproducible R code are available online at https://osf.io/gxudp/ (accessed on 13 October 2021).

## Supplementary Materials

The following are available online at https://www.mdpi.com/article/10.3390/brainsci11101346/s1, Figure S1: Average adjacency matrix for the delta frequency band, Figure S2: Average adjacency matrix for the theta frequency band, Figure S3: Average adjacency matrix for the alpha frequency band, Figure S4: Average adjacency matrix for the beta frequency band, Figure S5: Average adjacency matrix for the gamma frequency band.
