# Supplementary material for: An Effortful Approach to Social Affiliation in Schizophrenia: Preliminary Evidence of Increased Theta and Alpha Connectivity during a Live Social Interaction
Source: Brain Sci. 2021 Oct 13;11(10):1346. doi: 10.3390/brainsci11101346 (PMC8534160; doi:10.3390/brainsci11101346)

Supplementary Materials for:

An Effortful Approach to Social Affiliation in Schizophrenia: Preliminary Evidence of Increased Theta and Alpha Connectivity During a Live Social Interaction

Figure S1

Average Adjacency Matrix for the Delta Frequency Band

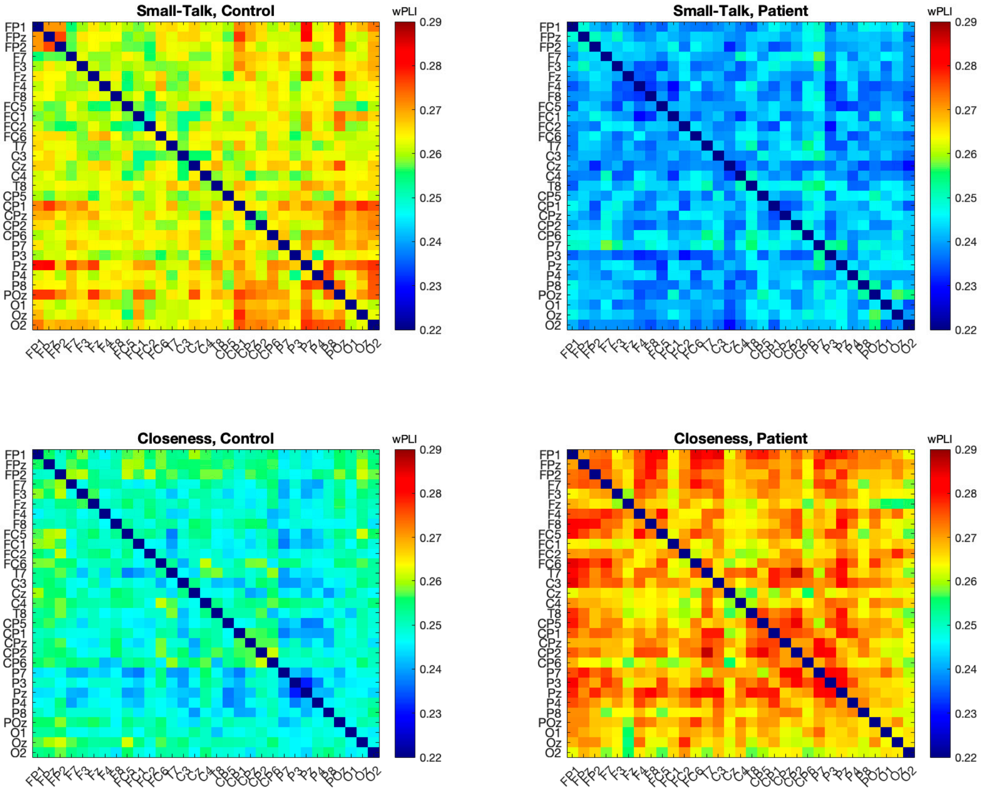

Figure S2

Average Adjacency Matrix for the Theta Frequency Band

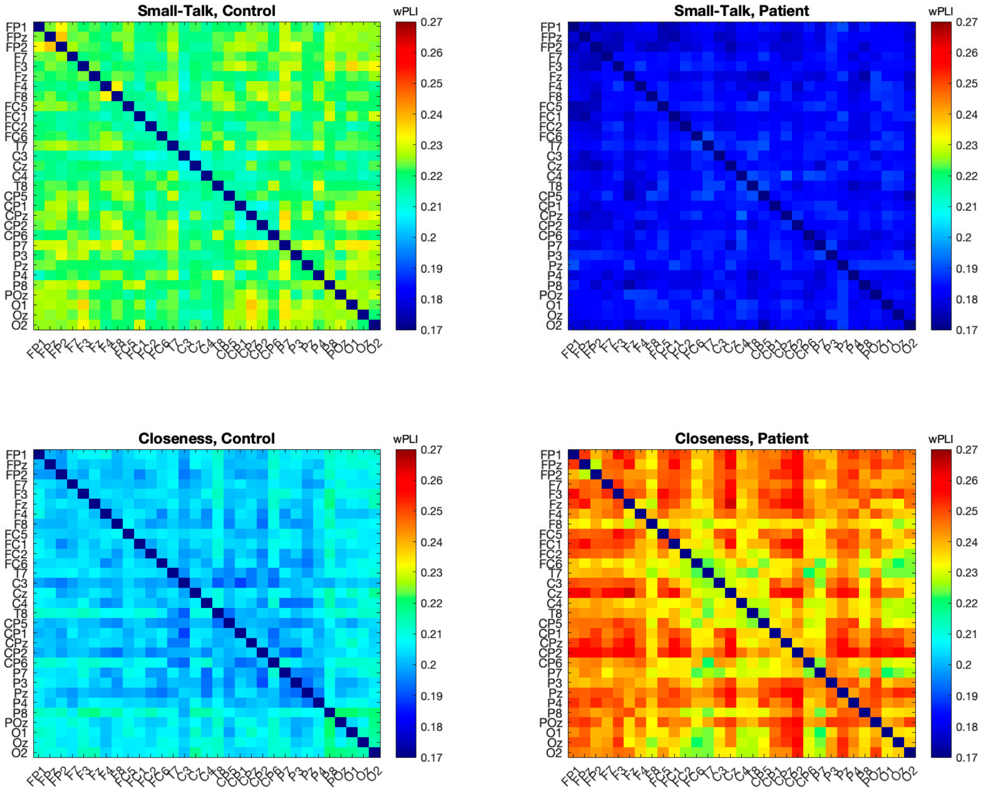

Figure S3

Average Adjacency Matrix for the Alpha Frequency Band

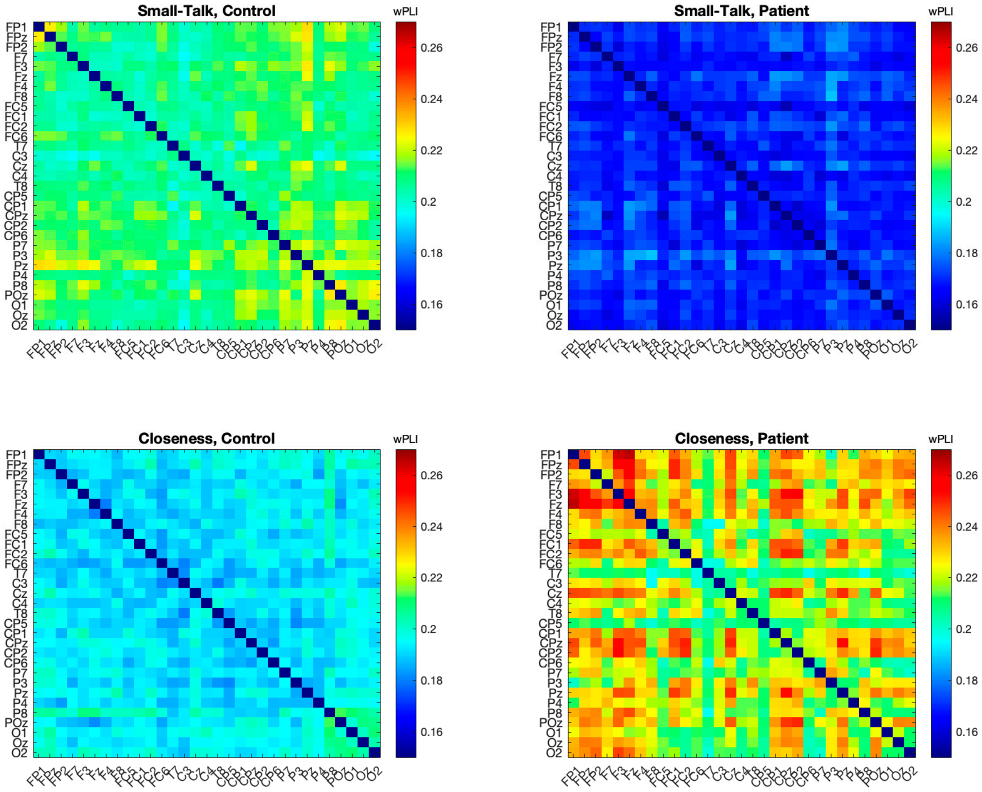

Figure S4

Average Adjacency Matrix for the Beta Frequency Band

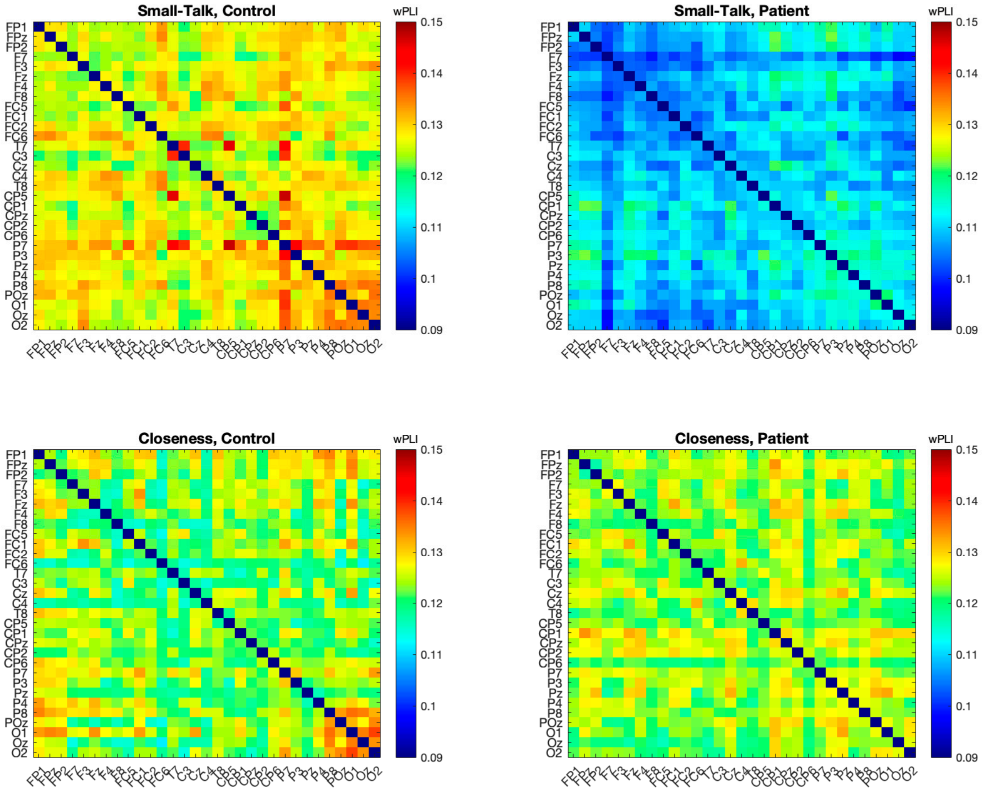

Figure S5

Average Adjacency Matrix for the Gamma Frequency Band

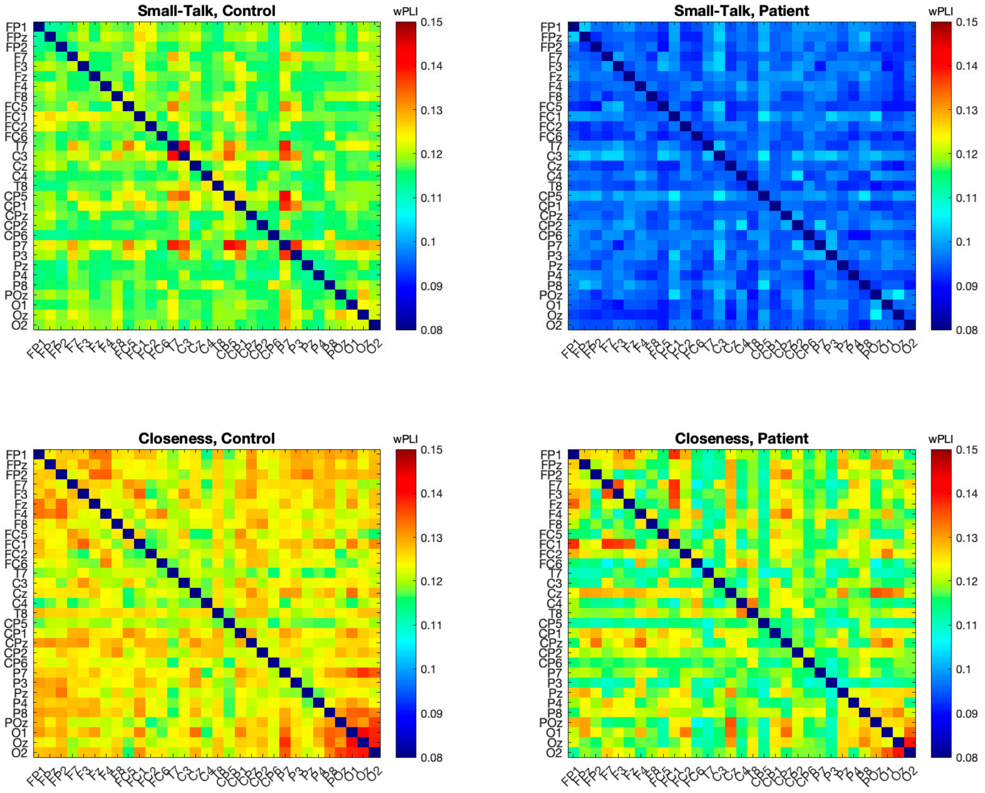

Supplement: Supplementary file 1 [file brainsci-11-01346-s001.zip › brainsci-1366739-supplementary.pdf]
